# Defining Consensus‐Based Components for Integrating Dietitians Into Primary Dental Care for Paediatric Populations: A Delphi Study

**DOI:** 10.1111/jhn.70305

**Published:** 2026-07-12

**Authors:** Lauren Hallewell, Raul Bescos, Zoe Brookes, Robert Witton, Patricia Casas‐Agustench

**Affiliations:** ^1^ School of Health Professions, Faculty of Health University of Plymouth Plymouth UK; ^2^ Peninsula Dental School, Faculty of Health University of Plymouth Plymouth UK

**Keywords:** consensus, Delphi, dietetics, integration, primary care dentistry

## Abstract

**Background:**

Diet is a modifiable risk factor linking oral and systemic health, contributing to dental caries and weight‐related outcomes (including underweight, overweight and obesity) in the paediatric population. However, dietary advice provided by dental professionals in primary dental care is often brief and sugar‐focused, representing a missed opportunity to address shared risk factors. Integrating dietitians into primary dental care may support more holistic paediatric preventative care.

**Objective:**

To develop expert consensus on the components required to integrate dietitians into primary dental care to address diet as a common risk factor in paediatric oral health and weight‐related outcomes.

**Methods:**

A three‐round online Delphi study was conducted with dietitians and dental professionals, followed by patient and public involvement (PPI) consultation with caregivers and children and young people. Round 1 open‐text responses were thematically analysed and synthesised into 158 statements. In Rounds 2 and 3, experts rated statements using a 7‐point Likert scale. Consensus agreement was defined *a priori* as ≥ 70% agreement (ratings of 6 or 7) and < 15% disagreement (ratings of 1 or 2) across the panel and within both professional groups. Results were summarised using descriptive statistics.

**Results:**

Thirty‐six, twenty‐eight, and twenty‐five experts completed Delphi Rounds 1, 2 and 3, respectively. Round 1 generated 158 statements, of which 90 achieved full consensus agreement by Round 3. Full consensus agreement was reached on safeguards for discussing weight‐related outcomes sensitively, referral criteria for oral‐health dietary support, training and skill development, organisational structures for collaboration, communication pathways and role clarification. No single approach to identifying weight‐related outcomes met the predefined agreement threshold and remained exploratory.

**Conclusion:**

This study identified consensus‐based components for integrating dietitians into primary dental care, providing a practical foundation to inform the design and piloting of integrated dietetic‐dental care models with potential to improve oral health and broader child health outcomes.

## Summary

- Integrating dietitians into primary dental care may provide an early access point for dietetic support, particularly where families experience difficulty implementing oral‐health‐related dietary advice or where oral symptoms affect eating.
- Clear role delineation is needed, with dental professionals providing brief oral‐health focused dietary advice and identifying when referral is appropriate, and dietitians delivering tailored, behaviourally informed dietary support.
- Joint training and regular case‐based discussions can support consistent messaging and strengthen interprofessional relationships.
- Effective integration requires defined referral pathways and shared systems, including shared patient records, support from local dietetic teams and dedicated time to develop professional relationships.
- Dietitians could address diet as a common risk factor for paediatric oral health weight‐related outcomes; however, approaches to identifying weight‐related concerns in dental settings remain uncertain and require further research.

## 1 Introduction

Oral and systemic health are closely connected and share common determinants, particularly diet. Dental caries (tooth decay) is the most prevalent non‐communicable disease, affecting 510 million children with deciduous (baby) teeth worldwide [1]. In children, body mass index (BMI) exhibits a U‐shaped association with dental caries, with higher risk among those living with underweight, overweight or obesity [2, 3]. When interpreted using age‐ and sex‐adjusted charts, BMI provides a practical indicator of paediatric weight‐related outcomes, although it should be considered alongside the wider clinical context [4]. Diet is modifiable and influences both dental caries susceptibility and weight‐related outcomes, including underweight, overweight, obesity [5].

Childhood is characterised by ongoing growth and development, representing a critical window for establishing dietary patterns that track into adulthood and influence health outcomes [6]. Dietary behaviours are influenced by caregiver choices and increasing child autonomy, making it a crucial stage for early intervention and highlighting the need to consider both the child and family context [7]. Primary dental care offers repeated contact with children and families and may provide opportunities to identify oral health‐ and/or weight‐related dietary concerns early [8, 9]. However, preventive dietary and weight‐related conversations led by dental teams are often constrained by appointment time, training, professional confidence, and limited referral pathways [10]. These barriers limit the ability of dental teams to provide sustained dietary support beyond brief advice [11]. Together, these factors make childhood a particularly important and timely focus for integrating dietetic expertise within dental care.

Dietitians are registered healthcare professionals who assess, diagnose, and manage dietary and nutritional problems across the life course using evidence‐based approaches, including tailored dietary support and behaviour change techniques [12]. Integrating dietitians into primary dental care may offer a pragmatic, patient‐centred approach to addressing shared dietary risk factors across oral and systemic health, extending preventive care beyond brief dental dietary advice [12, 13]. This aligns with the Common Risk Factor Approach (CRFA), which emphasises coordinated action on shared determinants of health [14, 15].

Although UK health policy emphasises prevention and workforce integration [16, 17, 18], there remains no structured model for embedding dietetic expertise within primary dental care, despite calls for stronger interprofessional collaboration between dietitians and dental professionals [19, 20]. Existing initiatives, such as dietetic internship placements in paediatric dental clinics, suggest potential value but lack a framework for routine clinical integration, standardised referral criteria, defined communication pathways, or role delineation [21, 22]. Barriers to interprofessional working include competing clinical priorities, inconsistent advice, and limited awareness of each other's professional scope of practice [23].

Therefore, this study aimed to develop expert consensus on the components required to integrate dietitians into primary dental care to address diet as a common risk factor in paediatric oral health and weight‐related outcomes.

## 2 Methods

### 2.1 Study Design

A three‐round online Delphi study was conducted to achieve expert consensus using anonymised surveys and controlled feedback, consistent with established Delphi methodology [24, 25]. This structured, iterative approach allows diverse professional groups to contribute while minimising dominant voices or groupthink [24].

The Delphi process followed classical methodological guidance [25] and comprised: (1) a qualitative round to generate statements; (2) a quantitative round to rate agreement and establish consensus on the statements; and (3) a second quantitative round to re‐rate non‐consensus statements with individualised feedback. Surveys were administered using JISC Online Surveys (version 3, Jisc, Bristol, UK) between May and August 2025, with approximately 3 weeks between rounds. A final consensus meeting with experts in September 2025 was used to contextualise findings.

A patient and public involvement (PPI) consultation was subsequently conducted (November–December 2025) to contextualise and support interpretation of the Delphi results. The PPI consultation was not part of the Delphi consensus process and did not contribute to consensus generation or modification of consensus outcomes. Rather, it was undertaken to contextualise the findings and provide patient‐centred validation of acceptability and relevance.

*Weight‐related outcomes* (underweight, overweight and obesity) are typically assessed within growth monitoring frameworks considering age‐ and sex‐specific height and weight [26, 27]. Therefore, the term *growth* was used as participant‐facing language. *Integration* referred to the physical co‐location of dietitians within primary dental care settings and coordinated care delivery.

### 2.2 Participants and Recruitment

Participants were recruited as an expert panel, using purposive sampling [25]. Eligible experts required ≥ 2 years' experience working with paediatric populations and relevant academic or clinical expertise in dentistry or dietetics; frequency of paediatric patient contact was not specified.

Dental professionals (dentists, hygienists, therapists, dental nurses), were recruited from four primary dental care settings where dental students are trained across South West England, collectively providing approximately 6260 appointments annually to 2500 children. Posters were presented in staff areas and emails were circulated via the gatekeeper.

Dietitians were recruited through the British Dietetic Association sharing posters through their communication channels, and targeted outreach to individuals with recognised expertise in paediatric dietetics, oral health, or weight management; identified through publicly available professional roles and recent publications. Experts included paediatric dietitians, dietitians with routine paediatric involvement in broader clinical roles, and academics with relevant research expertise. Dietitians were recruited from hospital, private practice, and community settings, reflecting typical locations of dietetic practice in the UK.

Students, non‐clinical staff, and professionals without direct involvement in patient‐facing care or peer‐reviewed research were excluded. Dietetic support roles were excluded to ensure inclusion of registered professionals with decision‐making responsibilities.

A target of 50 experts (25 dietitians, 25 dental professionals) was set to enable profession‐specific comparisons, while maintaining an anticipated retention rate of ≥ 70% across Delphi rounds [24, 28, 29]. Panels of 15–30 experts are commonly considered sufficient for Delphi consensus [25, 29, 30].

Interested individuals received an online information sheet, consent form, eligibility screener and demographic questionnaire. Eligible respondents were invited to participate in Round 1. Demographic data was collected on professional role, years of experience, qualifications and practice sector.

Ethical approval was obtained from the University of Plymouth, Faculty of Health Research Ethics and Integrity Board (Reference: 5566). Experts provided electronic informed consent prior to participation and were assigned unique identification codes to enable controlled feedback while maintaining anonymity during data analysis.

### 2.3 Setting and Target Population

This study focused on paediatric populations, defined as children and young people (CYP) under 18 years of age, in line with UK research guidance (children < 16 years; young people aged 16–17 years) [31]. Although definitions may vary across clinical services and settings, this threshold was used for consistency. An exemplar primary dental care setting where dental students are trained was used to provide contextual framing and support interpretation of survey items. Responses were not restricted by service configuration, allowing development of an idealised, transferable model of care.

### 2.4 Theoretical Framework and Survey Development

The study followed Conducting and Reporting Delphi Studies (CREDES) reporting guidelines (Supporting Information S1: Table 1) [32].

**Figure 1:**
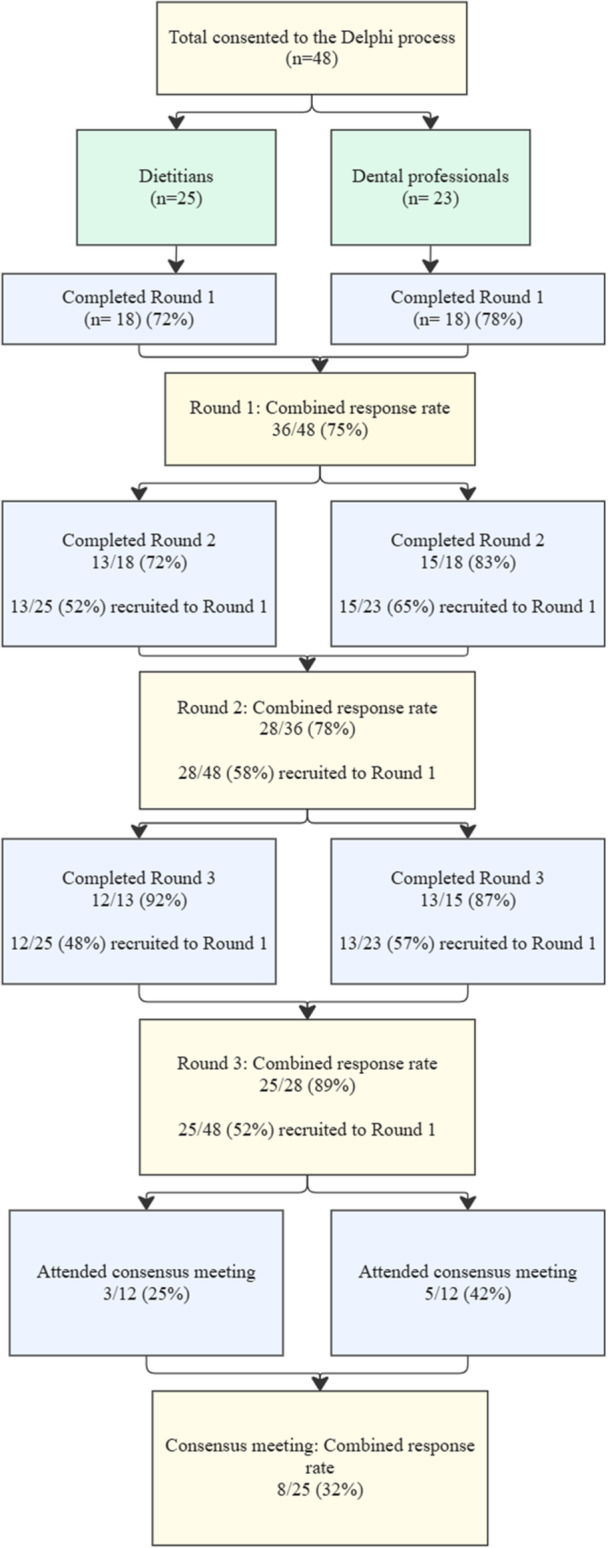
Flow diagram of Delphi participants.

Survey development was informed by the BCW for intervention design [33]. This study focused on Stage 1 of the BCW (understanding the behaviour) [33] (Supporting Information S1: Methods S1 and Figure 1), which was used to define the behavioural problem and identified two clinical target behaviours; *sensitive approaches to supporting weight‐related concerns*, and *supporting families to implement oral‐health‐related dietary advice* and four enabling conditions: *training and skill development*; *supportive structures for interdisciplinary collaboration*; *communication pathways*; and *role clarity and responsibilities* (Supporting Information S1: Table 2). These were informed by earlier research phases, including a scoping review [10] and qualitative interviews [34], alongside existing literature, and remained unchanged throughout the Delphi process.

Initial behavioural specification (Supporting Information S1: Table 3) and COM‐B mapping (Supporting Information S1: Table 4) were provisional, undertaken at a high level in the pre‐Delphi phase, and informed by existing literature to structure the problem and identify gaps [11, 33]. The Delphi process was then used to refine and specify these behaviours and enabling conditions (e.g., who performs the behaviour, with whom, when and where it would occur [11, 33]), with COM‐B mapping updated to reflect consensus findings (Supporting Information S1: Table 8).

Study aims and survey content were presented at the beginning of each survey, with questions structured around the clinical target behaviours and enabling conditions.

## 3 Delphi Process

### 3.1 Round 1: Statement Generation

#### 3.1.1 Survey Design

The Round 1 survey was iteratively developed, externally reviewed and piloted with a dietitian and two dental professionals. A qualitative survey comprising 18 open‐ended questions explored how the target behaviours and enabling conditions could be configured in practice. Each behaviour/condition was presented on a separate page, with 1–5 questions per page, designed to elicit behavioural specification. Round 1 questions are provided in Supporting Information S1: Table 5.

The behaviour relating to *sensitive approaches to weight‐related concerns* was intentionally framed broadly to reflect uncertainty regarding the most appropriate and preferred identification approaches, informed by qualitative interview findings (unpublished data) and to allow multiple perspectives to emerge.

#### 3.1.2 Qualitative Analysis

Qualitative responses from Round 1 were analysed using a hybrid inductive‐deductive thematic analysis in NVivo 14 (QSR International, Melbourne, Australia). The multidisciplinary research team included dietetic, nutrition and dental expertise, and data interpretation was discussed within the team throughout the study to minimise potential bias. A priori categories based on the target behaviours and enabling conditions were used to provide a deductive analytical framework, while inductive coding was conducted by the first author to generate data‐driven codes reflecting the original meaning of experts' responses. Codes were iteratively reviewed and refined in collaboration with the last author. To preserve the breadth of expert input, all concepts were retained during coding, including those raised by a single participant, to ensure minority perspectives were represented. Themes were then synthesised into concise statements for quantitative rating in subsequent Delphi rounds.

### 3.2 Rounds 2 and 3: Rating and Consensus

#### 3.2.1 Survey Design

These rounds were not piloted but reviewed internally. Statements from Round 1 responses were rated using a 7‑point Likert scale (higher scores indicating stronger agreement). Statements not meeting the full consensus threshold in Round 2 were re‐rated in Round 3, alongside feedback (median rating, overall and profession‐specific distribution of agreement, and participants' individual prior responses).

Statements not reaching consensus criteria after Round 3 were not included as consensus items but were used to inform the interpretation of areas of uncertainty.

#### 3.2.2 Quantitative Analysis

Quantitative data from Rounds 2 and 3 were analysed using Excel (Microsoft Corporation, Redmond, WA, USA) and MATLAB (R2025a; MathWorks, Natick, MA, USA). Consensus definitions were specified *a priori* and followed established Delphi standards (Table 1). Full consensus agreement required ≥ 70% of respondents to rate a statement 6 or 7, and < 15% to rate 1 or 2, both overall and within each professional group [35, 36]. This conservative threshold reflects the principle that items should be supported by a clear majority, with minimal dissent [35, 36]. Stability between Rounds 2 and 3 was assessed using the Wilcoxon signed‐rank test for paired ordinal data, with statistical significance set at *p* < 0.05. A conceptual care pathway was developed to support interpretation.

**Table 1:** Consensus definitions.

Domain	Definition/Rule
Agreement	≥ 70% of responses rated 6–7 and < 15% rated 1–2
Disagreement	≥ 70% of responses rated 1–2 and < 15% rated 6–7
Neutral	≥ 70% of responses rated 3–5
Overall consensus	Agreement, disagreement or neutral achieved in the full panel (all professions combined)
Profession‐specific consensus	Agreement, disagreement or neutral achieved within each profession (dietitians and dental professionals) analysed separately
Full consensus (primary criterion)	Agreement, disagreement or neutral achieved both overall and within both professions.

### 3.3 Consensus Meeting

Experts completing Round 3 were invited to a 60‐min online consensus meeting in September 2025 using Microsoft Teams (Microsoft Corporation, Redmond, WA, USA). The meeting reviewed items that met the pre‐defined consensus criteria and explored reasons for persistent divergence on non‐consensus statements between professional groups. No re‐voting occurred; and consensus outcomes were derived exclusively from the survey rounds.

### 3.4 PPI Consultation

A PPI consultation with caregivers and CYP aged 12–17 years was conducted after completion of all Delphi rounds to explore the acceptability and perceived appropriateness of patient‐facing components, in line with Picker Institute guidance [37]. The consultation focused on the two clinical target behaviours, as these were most relevant to families; enabling conditions were excluded as focused on clinical and interprofessional systems.

Ethical approval was obtained from the University of Plymouth, Faculty of Health Research Ethics and Integrity Board (Reference: 6669). Contributors were recruited during dental appointments across the same four primary dental care settings used for dental professional recruitment over a 1‐month period (November–December 2025). Caregivers of CYP of any age and CYP aged 12–17 years were eligible, with no additional eligibility criteria applied. CYP participation occurred via caregiver–child dyads rather than independent recruitment. As this activity was conducted as a PPI consultation rather than a formal research study, no predefined sample size was set, and recruitment was undertaken pragmatically.

Caregivers provided electronic consent for themselves and, where applicable, for their CYP, and CYP provided electronic assent prior to participation. The age range of 12–17 years was selected to ensure contributors could engage meaningfully with potentially sensitive topics and aligns with the study definition of CYP. Contributors were informed that participation and responses would not affect their care. Participation was voluntary and anonymised, with no identifiable data collected, to minimise perceived pressure and potential response bias.

An online survey (JISC Online Surveys) incorporating both closed‐response and free‐text questions was used, enabling the collection of quantitative and qualitative feedback. Survey items were derived from the final Delphi outputs and adapted for accessibility, focusing on the acceptability and preferred approaches to identifying weight‐related concerns and referral for oral‐health‐related dietary support. The PPI consultation was conducted as a single‐stage activity, and contributors did not provide ongoing or iterative feedback.

Responses were summarised descriptively, with free‐text responses reviewed to identify key themes. PPI findings were used to contextualise and support the interpretation of the Delphi results rather than to inform or modify expert consensus outcomes.

## 4 Results

### 4.1 Expert Panellists' Characteristics

Of 48 consenting experts, 36 completed Round 1, 28 Round 2, and 25 Round 3 (Figure 1). Only experts completing the preceding round progressed to the next. The final Delphi panel comprised 13 dental professionals and 12 dietitians. The achieved panel size (*n* = 25) aligns with recommended Delphi panel sizes, and retention across rounds (69%) is comparable to similar studies [24, 25, 28, 29, 30].

**Figure 2:**
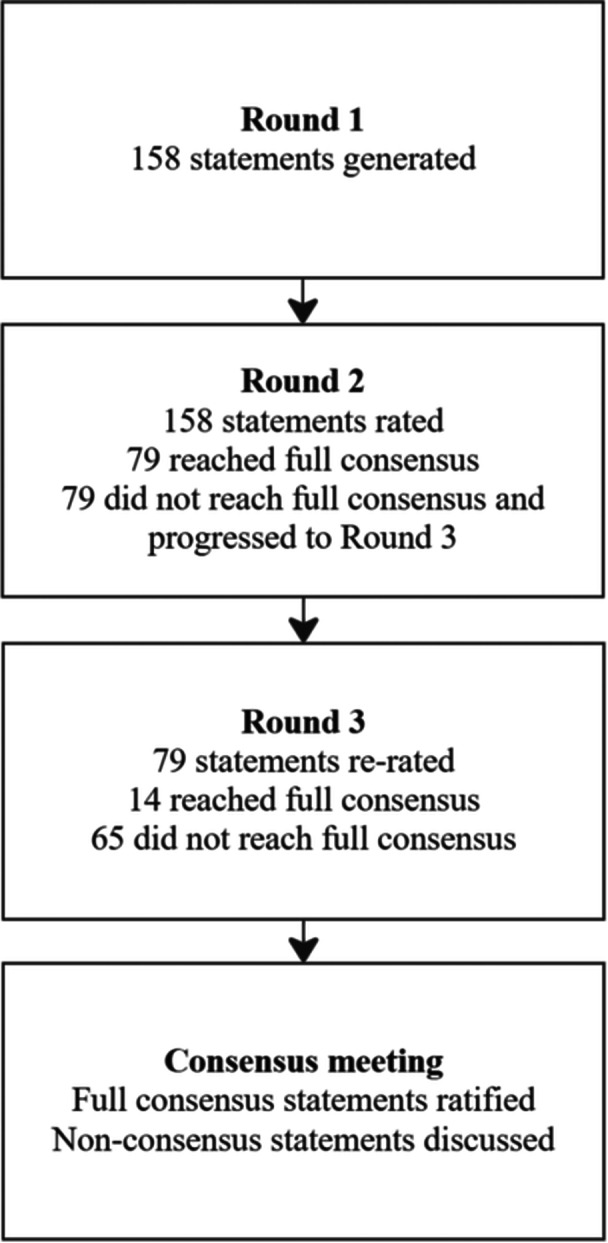
Delphi process across three survey rounds and a consensus meeting. This figure summarises progression across the three‐round Delphi process and the subsequent consensus meeting.

Among dental professionals completing all three rounds, 46% (*n* = 6) were general dentists, 23% (*n* = 3) dental therapists, 23% (*n* = 3) dental hygienists and 8% (*n* = 1) a dental nurse; 54% (*n* = 7) had > 25 years' experience. Dietitians represented hospital, community, academia (teaching), research, private practice and combined clinical settings; 42% (*n* = 5) had > 25 years' experience. Further details are provided in Supporting Information S1: Figure 2.

### 4.2 Overview of Consensus Over Delphi Rounds

Round 1 open‐text responses generated 158 statements. Following analysis, overlapping responses were refined and one highly variable item was reformulated as a model‐choice question. In Round 2, 79 statements achieved full consensus, while 79 remained unresolved across 11 questions. Round 3 produced a further 14 full consensus statements, yielding 93 consensus statements overall (90 agreement, 2 neutral, and 1 disagreement). Sixty‐five statements remained without full consensus, indicating persistent uncertainty (Figure 2). The stability of responses between Rounds 2 and 3 suggested robustness of the non‐consensus findings, with only 2 of 79 statements demonstrating statistically significant differences. Full consensus statements are presented in Supporting Information S1: Table 6.

### 4.3 Consensus by Target Behaviours and Enabling Conditions

#### 4.3.1 Sensitive Approaches to Supporting Weight‐Related Concerns

**Figure 3:**
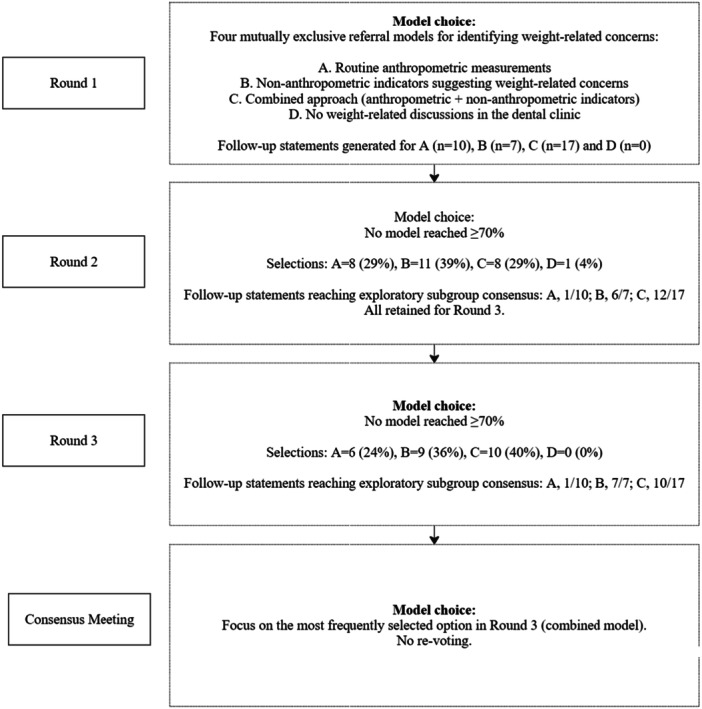
Progression of the model choice item for identifying weight‐related concerns across Delphi rounds. Four mutually exclusive models were generated from Round 1 responses and presented in Rounds 2 and 3. Model A: *Routine anthropometric measurements*: Dietitian‐led routine anthropometric measurements (height and weight, with BMI plotted) for all children attending the clinic; however, participation would be optional for families. Model B: *Non‐anthropometric indicators suggesting weight‐related concerns:* Concerns would be identified through non‐anthropometric indicators, rather than anthropometric measurements. These could include dietary records, discussions with dental professionals, or concerns raised by a caregiver or the child regarding weight‐related. Model C: *Combined approach (routine anthropometric measurements and non‐anthropometric indicators)*: This option would incorporate anthropometric measurements, while also identifying concerns based on non‐anthropometric indicators. Model D: *No discussion of weight‐related in the clinic*: Dietitians would not address weight‐related concerns within the dental clinic setting. No model reached the predefined agreement threshold (≥ 70% selection of a single model overall and across both professions). Follow‐up statements were analysed within model‐specific subgroups, with ≥ 70% agreement interpreted as exploratory subgroup consensus.

This target behaviour addressed both the identification and sensitive discussion of weight‐related concerns within primary dental care. Round 1 responses on identification were highly variable and contextual. To reflect this heterogeneity and to avoid prematurely collapsing distinct perspectives into single statements, four mutually exclusive referral models were derived and tested in subsequent rounds using a model‐choice item (Figure 3). Each model selection was followed by model‐specific follow‐up questions rated on a 7‐point Likert scale.

Across Rounds 2 and 3, expert views diverged, and no referral model reached the pre‐defined agreement threshold (defined as ≥ 70% selection of a single model overall and within each professional group). The combined approach (Model C) was the most frequently selected in Round 3 (40%) but did not meet the agreement threshold. No experts selected Model D in Round 3.

Experts selecting Model C, 10 of 17 follow‐up statements reached the consensus agreement threshold (≥ 70% selecting 6 or 7) within that subgroup and were interpreted as exploratory subgroup consensus (Supporting Information S1: Table 7). As these items were rated only by experts selecting this model, agreement reflects within‐group preferences rather than full‐panel and stratified by profession consensus. These statements reflected preferences for both anthropometric measurements at new patient registration, alongside referral based on non‐anthropometric indicators including caregiver or child concerns about weight, and dietary discussions with the dental team indicating potential weight‐related risks. Therefore, this is an exploratory finding, not to be interpreted as practice recommendations.

Despite the lack of consensus on a single identification model, 15 of 16 statements on sensitive handling of weight‐related concerns reached full consensus agreement, including dietitian‐led discussions and framing weight‐related discussions within a holistic health context (Supporting Information S1: Table 6).

#### 4.3.2 Supporting Families to Implement Oral‐Health‐Related Dietary Advice

Full consensus agreement was reached on referral criteria for dietetic support, namely when implementation of dental dietary advice had been unsuccessful, when oral symptoms affected eating behaviour, or when families requested additional support, regardless of disease severity.

#### 4.3.3 Enabling Conditions

Full consensus agreement was achieved on enabling conditions supporting interdisciplinary collaboration. *Training and skill development* were supported through hybrid training (self‐paced online modules with live or in‐person workshops), case discussions within team meetings and multidisciplinary team clinical supervision sessions. Full consensus agreement was also reached on *supportive structures* including shared care plans, digital notes accessible to both professions and annual review meetings to evaluate service impact, and dietitian access to community and hospital medical records. *Communication pathways* were agreed upon through shared digital patient records, supplemented by timely updates following significant changes in care and weekly documentation during periods of active involvement. Consensus was not reached on the use of phone calls for urgent cases and emails for routine communications. *Role clarity and responsibilities* achieved consensus agreement: dietitians should provide in‐depth dietary and/or weight‐related support, while dental professionals deliver brief preventive oral‐health dietary advice. Full consensus agreement was reached on boundaries for managing children already receiving dietetic care or requiring referral to specialist services. However, the required level of dietetic expertise did not reach consensus.

### 4.4 Synthesis of Findings

Consensus and exploratory findings were mapped to the BCW behavioural specification framework (Table 2) to define each target behaviour and enabling condition, resulting in the final Delphi‐informed specification. Findings were then interpreted in relation to the COM‐B model to inform understanding of behavioural determinants (Supporting Information S1: Table 8). This differed from the provisional specification and COM‐B mapping conducted prior to the Delphi process (Supporting Information S1: Tables 2–3).

**Table 2:** Delphi‐informed behavioural specification of target behaviours and enabling conditions.

Target behaviour summary	Who needs to perform the behaviours?	What do they need to do differently?	When and where should the behaviour occur?	How often should the behaviour occur?	With whom should the behaviour occur?
Sensitive approaches to support weight‐related concerns (*Clinical behaviour)*	Dietitians lead	Identify concerns based on anthropometric and non‐anthropometric risk factors; however, no single identification model reached consensus.^a^	May include anthropometric measures (e.g., BMI measured at new patient registration, increased monitoring for children with medical conditions) and non‐anthropometric indicators (e.g. use of caregiver/child concerns, dental professional identifies weight concern risk factors, recent measurements show rapid change and dietary risk factors), based on exploratory subgroup findings.^b^	In relation to the previous column.^a^	Child; family; dental professional; dietitian; external dietetic/specialist services where required.
		Support families in holistic discussion, using neutral language, visual aids, age‐appropriate discussions and written materials.			
			In a private room (within dental clinic).		
Supporting families to implement oral‐health‐related dietary advice (*Clinical behaviour)*	Dental professionals; dietitians; families.	Dental professionals provide initial dietary advice; dietitians provide tailored, behaviourally informed support.	Referral from dental professional when; oral symptoms affect eating, dental dietary advice is not implemented, families express confusion or difficulty with dental dietary advice and when families request support regardless of disease severity.	When referral criteria are met.	Child; family; dental professional; dietitian; external dietetic/specialist services where required.
Training and skill development *(Enabling condition*)	Dental professionals and dietitians.	Develop knowledge and skills in diet‐oral health links, behaviour change, impact of medical conditions on oral health and diet, how to balance oral‐health and diet advice, oral health risks in medically vulnerable children, ethical considerations in shared care, practical strategies of educating families, tailoring advice for families from a variety of cultural and socioeconomic backgrounds and principles of interprofessional teamwork and communication.	Workshops (in‐person and virtual), MDT clinical supervision sessions, integrating case discussions in meetings or huddles and hybrid approach of self‐paced modules and in‐person/clinical supervision sessions.	Was not asked in Delphi. Likely dependent on individuals and would require review and monitoring.	Dental professional and dietitian.
Supportive structures *(Enabling condition)*	Existing dental and dietetic care systems.	Implement systems to support collaboration including, referral pathways, access to medical records, dietetic mentor, connection to local dietetic teams, structured induction to dental clinic, connection to specialist dietetic teams, dental supervisor (for dietitian) and dedicated time for relationship building.	Embedded within primary dental service design and organisational processes with existing dietetic services.	Was not asked in Delphi. Likely dependent on contextual factors such as resources, case load of existing teams, etc.	Dental professionals; dietitians; external dietetic teams and wider healthcare services (not limited to primary care)
Communication pathways *(Enabling condition)*	Dental professionals and dietitians.	Place referral, share patient information, care plans and update using communication systems.	Shared digital patient records.	Communication when significant changes occur in care, use of flagging systems to alert to dietary issues and twice weekly updates during periods of active dietitian involvement.	Dental professionals and dietitians.
				Annual review meetings for evaluation of service.	
Role clarity and responsibility *(Enabling condition)*	Dental professionals and dietitians.	Maintain clear role boundaries: dietitians provide in‐depth dietary support, dental professionals provide brief oral‐health focused dietary advice, messages are consistent, all dietary advice provided by the dental team should align with the broader care plan agreed with the dietitian and dietitians should liaise with and refer to specialist services where appropriate and determine input following liaison.	During patient care, referral decisions and interdisciplinary collaboration.	Throughout care delivery.	Dental professionals; dietitians; external dietetic teams and wider healthcare services (not limited to primary care).

^a^ Model‐choice item where no single option reached the consensus threshold.

^b^ Exploratory subgroup findings based on participants selecting the most frequently endorsed model; not full‐panel consensus and should not be interpreted as practice recommendations.

**Figure 4:**
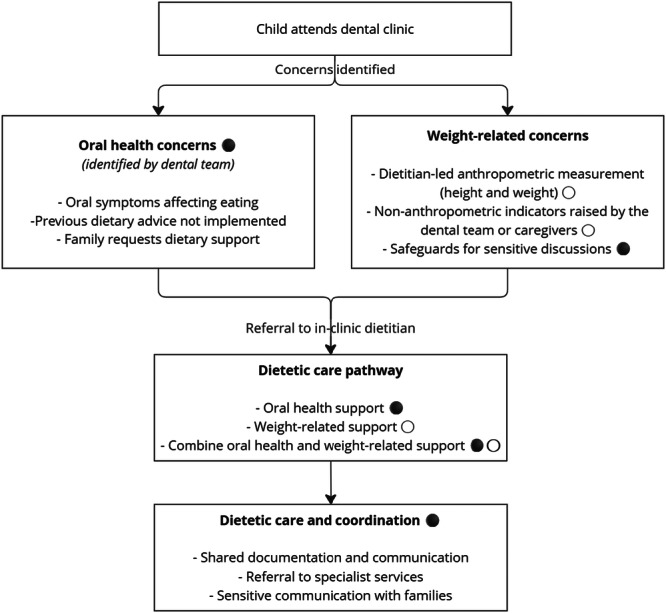
Conceptual care pathway. This figure synthesises Delphi‐derived consensus and non‐consensus statements into an illustrative conceptual care pathway. ● markers indicate components that achieved full consensus agreement. ○ markers denote exploratory elements where full consensus was not reached, with the most frequently selected approach to identifying weight‐related concerns presented (not practice recommendations). The figure illustrates conceptual relationships between components rather than an operationalised sequence of care and should not be interpreted as a validated clinical pathway.

A conceptual care pathway was developed to illustrate how consensus‐based components might be organised within primary dental care. It was based primarily on statements that achieved full consensus agreement and incorporates the most frequently selected model for identifying weight‐related concerns (exploratory). Therefore, weight‐related identification elements should be interpreted as hypotheses for future testing rather than practice recommendations. Visual markers indicate areas of uncertainty. The pathway is presented as a conceptual illustration of the findings rather than as a prescriptive clinical workflow (Figure 4).

### 4.5 Consensus Meeting Insights

The online consensus meeting (*n* = 8; five dental professionals, three dietitians) provided qualitative context for unresolved items. Divergence appeared to reflect variable familiarity with professional scopes of practice. Experts emphasised the importance of mutual role understanding for successful integration.

### 4.6 Summary of PPI Consultation

Forty‐eight caregivers and five CYP provided feedback. Most (80%) caregivers and all CYP considered dietetic support focused on oral health as acceptable or very acceptable, with only one (2%) selecting not acceptable at all. Caregiver acceptance of oral‐health‐related referral criteria ranged from 67%–83%. These findings were broadly consistent with the Delphi results.

Views on identifying weight‐related concerns varied. Among caregivers, the combined approach, incorporating anthropometric measurements and non‐anthropometric indicators of weight‐related concerns, was selected most frequently (53%). CYP preferences were distributed across the available options: two preferred routine anthropometric measurements, two preferred the combined approach, and one preferred referral based on non‐anthropometric indicators suggesting weight‐related concerns. These findings aligned with the Delphi uncertainty.

## 5 Discussion

This study addresses a key gap between literature calls for interprofessional collaboration to address CRFA and the limited practical guidance on how such collaboration can be implemented in primary care dentistry. Unlike existing literature, which advocates collaboration in principle or largely describes barriers, this study provides a consensus‐based, theory‐informed specification of how dietetic‐dental integration could be delivered in practice, addressing diet as a common risk factor in paediatric populations.

### 5.1 Sensitive Approaches to Supporting Weight‐Related Concerns

A key finding was that uncertainty surrounding weight‐related care related not to whether it should be addressed, but to how it can be delivered appropriately. Although no single model reached agreement threshold, none selected excluding weight‐related discussions. The PPI consultation reflected similar variability, with only one selecting this should not be discussed in dental settings. This study identified alternative approaches to identifying weight‐related concerns beyond anthropometric measurements and introduced non‐anthropometric risk indicators as an identification mechanism, such as caregiver concerns, dietary diaries indicating higher risk. This extends the existing literature on weight management interventions in dentistry, which has predominantly focused on height and weight screening, BMI screening and weight discussions or counselling [38].

Routine anthropometric measurements in primary dental care could support early identification of children requiring weight‐related support and help address existing gaps in early childhood monitoring, such as the National Child Measurement Programme [4]. However, reliance on primary dental care settings introduces self‐selection bias, as only families accessing care are reached, potentially exacerbating inequalities among populations disproportionately affected by limited dental access, obesity and food insecurity [3]. Non‐anthropometric indicators, including caregiver concern and dietary risk factors, were also supported as referral triggers. Exclusive reliance on non‐anthropometric indicators may risk missed identification, as caregivers may not recognise overweight or obesity in their child, underestimate its health implications, or may not feel comfortable in talking about weight particularly around disordered eating behaviours [39, 40]. This interpretation reflects broader tensions in childhood weight‐related interventions, which require balancing early identification with concerns about stigma and psychological harm [41].

The consensus agreement on framing discussions within a holistic health context (rather than weight‐focused messaging) provides a clear direction for practice and aligns with stigma‐reducing approaches such as the Health At Every Size [42]. While much of the existing literature has focused on overweight and obesity, the weight‐related concerns considered in this study also encompass underweight concerns. Consequently, approaches to identification and referral should be sufficiently flexible to support children across the spectrum of weight‐related concerns. Accordingly, the identification of weight‐related outcomes incorporated within the conceptual care pathway should be interpreted as exploratory rather than ready‐to‐implement clinical recommendations.

### 5.2 Supporting Families to Implement Oral‐Health‐Related Dietary Advice

In contrast, consensus on referral criteria for oral‐health‐related dietary support provides an actionable contribution. Existing models of student dietetic involvement in dentistry primarily links consultations to caries risk assessment and sugar exposure scores [21]. This study extends these models by identifying broader criteria, including repeated difficulty implementing dental dietary advice, oral conditions affecting eating, and family‐initiated requests for support, regardless of disease severity. This reframes dietetic input from a reactive, risk‐based model to a preventive, needs‐based approach, representing a shift in how dietary care could be positioned within dentistry.

These findings align with the CRFA, recognising that oral health and obesity share common social and cultural determinants influencing dietary behaviour [43]. This provides a mechanism for integrating dietitians into routine care, addressing a recognised limitation in dental services where time and training constrain tailored dietary advice [10, 43, 44]. Concordance between Delphi and PPI findings strengthens confidence in the acceptability and relevance of these criteria.

### 5.3 Enabling Conditions

The study advances understanding of how interprofessional collaboration can be implemented in practice. While existing literature identifies educational needs, inconsistent advice and limited electronic shared record systems as barriers [23, 45], it often lacks detail on how these should be applied within integrated care. This study addresses this gap by identifying actionable components for dietetic‐dental collaboration, aligning with the Interprofessional Education Collaborative (IPEC) framework, which emphasise shared values, communication and teamwork as core competences [14]. While the IPEC does not specify structural requirements, it highlights the importance of shared frameworks, organisational practices and access to information that enable safe and effective interprofessional collaboration [14]. The findings extend the recommendation by Ong et al., regarding educational initiatives that enhance dietetic‐dental collaboration by specifying training content and delivery approaches [23]. Experts endorsed reciprocal learning through joint training, case reviews, supervision, shared records, online referral processes, and clear role delineation. Consistent with wider evidence, these findings indicate that effective interprofessional collaboration depends not only on workforce capacity but also on organisational systems that support coordination and continuity [23, 46]. Whilst the IPEC does not specify structural requirements, it highlights the importance of shared frameworks, organisational practices and access to information that enable safe and effective interprofessional teamwork [14].

Relational factors were also emphasised, including mentorship, structured induction, and protected time for relationship‐building. These align with broader integrated‐care literature showing that communication extends beyond documentation and information exchange; trust, transparency, tone, and responsiveness influence how clinicians engage in decision‐making and work together in practice [47]. These relational elements are reflected in IPEC competencies on values and ethics, which emphasise trust, empathy and mutual respect [14]. While care pathways may facilitate coordination, they cannot alone create the relational conditions required for effective collaboration [48].

### 5.4 Strengths, Limitations and Future Research

This study employed a theory‐informed Delphi process across two professional groups and incorporated PPI consultation to contextualise selected patient‐facing components. The findings extend existing literature by shifting the focus from whether collaboration between dietitians and dental professionals is beneficial to how such collaboration could be operationalised in practice. Accordingly, the study provides a structured, consensus‐based set of components to inform future refinement and piloting. The findings also have implications for service design, as the lack of consensus regarding dietetic expertise suggests that different integration models may be appropriate depending on available expertise and organisational context. In this respect, future economic evaluation will be important, as preventive approaches may require initial investment despite potential longer‐term health and system‐level benefits [49].

Several limitations should be acknowledged. The findings reflect expert opinion, which does not necessarily represent ‘correct’ answers. Some elements, particularly approaches to identifying weight‐related concerns, remain exploratory and indicate areas of uncertainty, highlighting priorities for future research to ensure that approaches are acceptable, equitable, and avoid potential harm.

A conservative consensus threshold was applied. Although this may have reduced the number of items reaching consensus, no items failed solely due to the disagreement criterion. Instead, non‐consensus reflected insufficient overall agreement and/or divergence between professional groups, suggesting genuine uncertainty rather than ‘false non‐consensus’. The PPI consultation was pragmatic and not designed for generalisability; recruitment in clinical settings may have introduced response bias, and prior experience with dietetic services was not assessed. Variation in professional context may also have influenced the findings, as dietitians contributed perspectives from multiple settings, whereas dental professionals reflected the primary care context in which integration is intended. In addition, the frequency of paediatric contact was not specified and may have varied between participants; however, the Delphi process was designed to capture expert perspectives informed by paediatric experience rather than to quantify exposure frequency. Findings may additionally be influenced by the geographical context, as dental professionals and PPI contributors were recruited from four primary dental care settings in the South West of England only. Furthermore, the consensus statements and proposed model were developed with UK healthcare professionals and may therefore not be directly transferable to other healthcare systems with different funding structures and care needs. The delivery of integrated‐dietetic care is likely to depend on local service structures, referral mechanisms, funding arrangements, workforce availability and access to shared digital systems. Consequently, adaptation and evaluation of the consensus statements may be required across different healthcare contexts.

## 6 Conclusion

This study provides a structured, theory‐informed foundation for integrating dietitians into primary dental care by specifying the clinical and enabling conditions required to support collaborative practice. By moving beyond calls for collaboration to defining how integration can be delivered in practice, the findings offer a clear foundation for future refinement, service development, piloting and evaluation, supporting a shift towards more coordinated and preventive dietary care for families.

## Author Contributions

**Lauren Hallewell:** conceptualisation, methodology, investigation, formal analysis, data curation, writing – original draft, writing – review and editing, visualisation, validation, project administration. **Raul Bescos:** conceptualisation, methodology, investigation, formal analysis, writing – review and editing, visualisation, validation, supervision, project administration. **Zoe Brookes:** conceptualisation, methodology, investigation, writing – review and editing, visualisation, validation, supervision, project administration. **Robert Witton:** conceptualisation, methodology, investigation, writing – review and editing, visualisation, validation, supervision, project administration. **Patricia Casas‐Agustench:** conceptualisation, methodology, investigation, formal analysis, data curation, writing – original draft, writing – review and editing, visualisation, validation, supervision, project administration.

## Funding

The authors have nothing to report.

## Ethics Statement

The study was approved by University of Plymouth Faculty of Health Research Ethics Committee (Ref: 5566, 6669).

## Conflicts of Interest

The authors declare no conflicts of interest.

## Supporting information


Supporting File

## Data Availability

The data that support the findings of this study are available on request from the corresponding author. The data are not publicly available due to privacy or ethical restrictions.
