# Supplementary material for: Defining Consensus‐Based Components for Integrating Dietitians Into Primary Dental Care for Paediatric Populations: A Delphi Study
Source: J Hum Nutr Diet. 2026 Jul 12;39(4):e70305. doi: 10.1111/jhn.70305 (PMC13358320; doi:10.1111/jhn.70305)
Supplement: Supplementary file 1 — Supporting File [file JHN-39-0-s001.docx]

**Supporting Information S1**

*Supplementary Methods*

- Supplementary Table 1. CREDES checklist
- Supplementary Methods S1. Stage One: Understanding the behaviour using the Behaviour Change Wheel for intervention design (pre-Delphi process)
  - *1.1 Define the problem in behavioural terms*
  - *1.2 Select target behaviours*
  - *1.3 Specify the target behaviours (provisional; refined via Delphi)*
  - *1.4 Identify what needs to change (COM-B mapping) (provisional; refined via Delphi)*
- Supplementary Figure 1. Behaviour Change Wheel stages for intervention design
- Supplementary Table 2. Target behaviours and enabling conditions
- Supplementary Table 3. Provisional specification of target behaviours and enabling conditions (pre-Delphi)
- Supplementary Table 4. Provisional COM-B mapping (identifying what needs to change) (pre-Delphi)
- Supplementary Table 5. Round 1 Questions

*Supplementary Results*

- Supplementary Figure 2. Additional demographic information
- Supplementary Table 6. Full consensus statements
- Supplementary Table 7. Subgroup-level exploratory consensus statements informing weight-related care
- Supplementary Table 8. Refined COM-B mapping (identifying what needs to change) following Delphi consensus

*Supplementary Methods*

**Supplementary Table 1. CREDES checklist**

| CREDES ITEM | Reported on Page |
| --- | --- |
| Rationale for the choice of the Delphi technique | |
| 1. *Justification.* The choice of the Delphi technique as a method of systematically collating expert consultation and building consensus needs to be well justified. When selecting the method to answer a particular research question, it is important to keep in mind its constructivist nature | 4 |
| 2. *Planning and process.* The Delphi technique is a flexible method and can be adjusted to the respective research aims and purposes. Any modifications should be justified by a rationale and be applied systematically and rigorously | 6-8 |
| 3. *Definition of consensus.* Unless not reasonable due to the explorative nature of the study, an a priori criterion for consensus should be defined. This includes a clear and transparent guide for action on (a) how to proceed with certain items or topics in the next survey round, (b) the required threshold to terminate the Delphi process and (c) procedures to be followed when consensus is (not) reached after one or more iterations | 8 |
| Study conduct | |
| 4. *Informational input.* All material provided to the expert panel at the outset of the project and throughout the Delphi process should be carefully reviewed and piloted in advance in order to examine the effect on experts’ judgements and to prevent bias | 6 |
| 5. *Prevention of bias.* Researchers need to take measures to avoid directly or indirectly influencing the experts’ judgements. If one or more members of the research team have a conflict of interest, entrusting an independent researcher with the main coordination of the Delphi study is advisable | 7 |
| 6. *Interpretation and processing of results.* Consensus does not necessarily imply the ‘correct’ answer or judgement; (non)consensus and stable disagreement provide informative insights and highlight differences in perspectives concerning the topic in question | 16 |
| 7. *External validation.* It is recommended to have the final draft of the resulting guidance on best practice in palliative care reviewed and approved by an external board or authority before publication and dissemination | To be reviewed in final study |
| Reporting | |
| 8. *Purpose and rationale.* The purpose of the study should be clearly defined and demonstrate the appropriateness of the use of the Delphi technique as a method to achieve the research aim. A rationale for the choice of the Delphi technique as the most suitable method needs to be provided | 4 |
| 9. *Expert panel.* Criteria for the selection of experts and transparent information on recruitment of the expert panel, socio-demographic details including information on expertise regarding the topic in question, (non)response and response rates over the ongoing iterations should be report | 4-5 |
| 10. *Description of the methods.* The methods employed need to be comprehensible; this includes information on preparatory steps (How was available evidence on the topic in question synthesised?), piloting of material and survey instruments, design of the survey instrument(s), the number and design of survey rounds, methods of data analysis, processing and synthesis of experts’ responses to inform the subsequent survey round and methodological decisions taken by the research team throughout the process | 4-8 |
| 11. *Procedure.* Flow chart to illustrate the stages of the Delphi process, including a preparatory phase, the actual ‘Delphi rounds’, interim steps of data processing and analysis, and concluding steps | 9-10 |
| 12. *Definition and attainment of consensus.* It needs to be comprehensible to the reader how consensus was achieved throughout the process, including strategies to deal with non-consensus | 9-11 |
| 13. *Results.* Reporting of results for each round separately is highly advisable in order to make the evolving of consensus over the rounds transparent. This includes figures showing the average group response, changes between rounds, as well as any modifications of the survey instrument such as deletion, addition or modification of survey items based on previous rounds | 9-12 |
| 14. *Discussion of limitations.* Reporting should include a critical reflection of potential limitations and their impact of the resulting guidance | 15-16 |
| 15. *Adequacy of conclusions.* The conclusions should adequately reflect the outcomes of the Delphi study with a view to the scope and applicability of the resulting practice guidance | 16 |
| 16. *Publication and dissemination.* The resulting guidance on good practice in palliative care should be clearly identifiable from the publication, including recommendations for transfer into practice and implementation. If the publication does not allow for a detailed presentation of either the resulting practice guidance or the methodological features of the applied Delphi technique, or both, reference to a more detailed presentation elsewhere should be made (e.g. availability of the full guideline from the authors or online; publication of a separate paper reporting on methodological details and particularities of the process (e.g. persistent disagreement and controversy on certain issues)). A dissemination plan should include endorsement of the guidance by professional associations and health care authorities to facilitate implementation | Throughout the manuscript. |

**Supplementary Methods S1. Stage One: Understanding the behaviour using the Behaviour Change Wheel for intervention design (Pre-Delphi process)**

This supplementary section describes the theoretical framework and processes used to understand the behaviours required for integrating dietitians into primary dental care.

The Behaviour Change Wheel (BCW) provides a structured theory-informed framework for intervention design, comprising three stages 1) understanding the behaviour, 2) identifying intervention options and 3) identifying content and implementation options (Supplementary Figure 1).^1^ This study focused on BCW Stage 1 (understanding the behaviour) and its four sub-stages to inform the subsequent Delphi process.

Stage 1 was conducted in two phases. First, Stage 1.1 (defining the problem in behavioural terms) and Stage 1.2 (selecting target behaviours) drew on semi-structured interviews (unpublished data), scoping review^2^ and existing literature to define the behavioural problem and identify target behaviours and enabling conditions. These components were established pre-Delphi process and remained unchanged throughout.

Second, Stage 1.3 (specifying target behaviours) and Stage 1.4 (identifying what needs to change using COM-B) were developed provisionally prior to the Delphi process, at a high level based on the literature only. Due to limited evidence and absence of stakeholder input, these were intentionally under specified. These elements were subsequently refined and specified through the Delphi process, with final outputs presented in Table X (main manuscript) and Supplementary Table 8. The provisional mapping provided a structured, theory-informed starting point for the Delphi process, enabling systematic refinement and specification of behaviours and enabling conditions based on expert consensus.

Importantly, only Stage 1.3 and Stage 1.4 were provisional; Stage 1.1 and Stage 1.2 did not change following the Delphi process.

***1.1 Define the problem in behavioural terms***

Defining the behavioural problem was informed by two complementary sources:

- A scoping review, informed by the Theoretical Domains Framework, identifying barriers and facilitators to dietary practices in paediatric dental care
- Qualitative interviews, informed by the Theoretical Framework of Acceptability, exploring perspectives on integrating dietitians into dental settings.

These data were supplemented by relevant literature to contextualise and interpret findings and synthesised to define the behavioural problem and inform selection of target behaviours, following established approaches to theory-informed intervention design.^3^

In current primary dental care systems serving paediatric populations, professionals are not consistently enabled to engage in or sustain effective dietary assessment and behaviour-change conversations with families beyond a narrow oral-health focus, despite widespread recognition of diet’s role in both oral and systemic health.^2,4,5^ Dental professionals may have limited nutritional training and confidence (*capability*), while operating within environments characterised by time constrains, unclear referral pathways and a lack of dietetic infrastructure (*opportunity*).^2^ Motivation may be further undermined by ambiguity around professional roles and inconsistent reinforcement.^2,6^

At the same time, the preventive value of dietetic input is widely acknowledged for its preventive role in systemic and oral health, yet practical mechanisms for delivering such care in dental settings remain undefined.^7–10^ Acceptability of such integration depends on supportive structures that safeguard emotional sensitivity in weight-related and dietary discussions, delineate professional boundaries, and ensure practical implementation considerations within routine care. The absence of an agreed model for dietetic integration contributes to fragmented dietary messaging, inconsistent caregiver support and missed opportunities for early prevention.^11^

Collectively, this analysis defines the behavioural problem as a system-level misalignment between professional capability, organisational opportunity and relational acceptability, indicating the need of reconfigured care processes, roles and communication systems to create the conditions for integrated dietetic practice within paediatric dental care.

***1.2 Select target behaviours***

Following BCW guidance, target behaviours were identified from the identified behavioural problem, which highlighted a range of actions and system-level processes relevant to improving dietary practices in paediatric dental care.

These candidate behaviours were reviewed and prioritised using established BCW criteria, including their potential impact on the behavioural problem,, feasibility within primary dental care, interaction with other behaviours and measurability.^12^ This process involved iterative consideration of how behaviours clustered and influenced one another, rather than treating them as discrete actions.

Through this process, two clinical behaviours (direct patient-facing practices) and four enabling conditions (organisational and system-level processes required to support those behaviours) were identified as priorities for further exploration within the Delphi process (Supplementary Table 2).

The inclusion of both behavioural and enabling components reflects the multi-level nature of integrated care, in which individual professional actions are dependent on supportive organisational structures and systems. This dual-level framing reflects the multi-level nature of integrated care, in which individual professional actions are closely interconnected with organisational structures and systems.^13^ This ensured that downstream specification (Stage 1.3) and COM-B analysis (Stage 1.4) captured both individual and contextual determinants of behaviour.

***1.3 Specify the target behaviours (provisional, later refined via Delphi)***

Target behaviours and enabling conditions were provisionally specified prior to the Delphi process using BCW behavioural specification principles, outlining who performs the behaviour, what they do and in the context in which it occurs.^12^

Some elements of behavioural specification, particularly timing and frequency, were intentionally left under-specified at this stage, due to limited evidence and absence of stakeholder input. These aspects were subsequently refined through the Delphi process, allowing detailed configuration of behaviours to be informed by expert consensus rather than predetermined assumptions.

***1.4 Identify what needs to change (COM-B mapping) (provisional, refined via Delphi)***

The COM-B model was applied to provisionally map potential determinants of the target behaviours and enabling conditions (Supplementary Table 3), encompassing capability, opportunity and motivation.^1^

This provisional mapping was informed by prior empirical work and the literature and represents a theory-informed preliminary analysis, rather than findings derived from the Delphi process. These determinants were subsequently refined and specified through Delphi consensus.

The preliminary analysis suggested a multi-level pattern of influences:

- *Capability*: Dietitians were expected to possess knowledge and skills required (e.g., anthropometry assessment, dietary counselling), although knowledge of oral-systemic health links may require further development.^14,15^
- *Opportunity*: Social and physical opportunity were anticipated to be key constraints, including limited time, absence of shared systems and unclear integration processes.
- *Motivation*: Literature suggests motivation may be influenced by professional identify, perceived role legitimacy, interprofessional trust and beliefs about preventive care. ^16^

**Supplementary Figure 1. Behaviour Change Wheel stages for intervention design**

*Adapted from Michie, Atkins, and Gainforth (2016).*^1^

*This figure illustrates the staged BCW process for intervention design. This study focused on Stage 1 (understanding the behaviour). Prior to the Delphi process, Stages 1.1 and 1.2 were defined based on the literature, while Stages 1.3 and 1.4 were provisionally mapped. The Delphi process subsequently refined and specified the target behaviours (Stage 1.3) and identified what needs to change (Stage 1.4).*


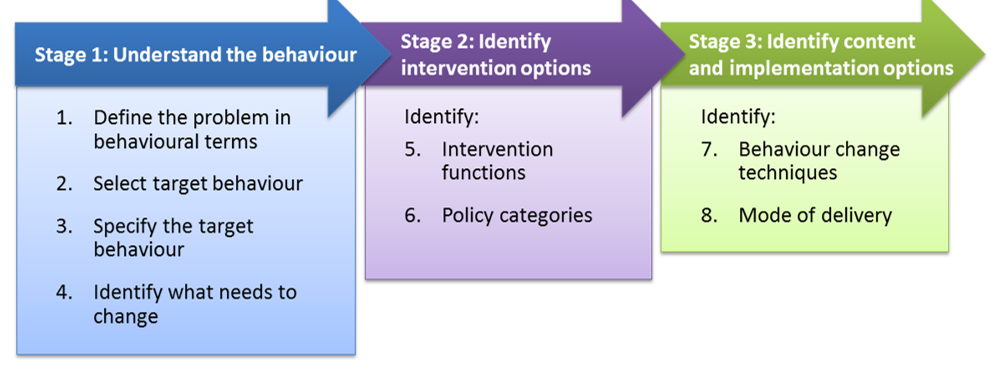


**Supplementary Table 2. Target behaviours and enabling conditions**

| Clinical Behaviours | Enabling conditions |
| --- | --- |
| Sensitive approaches to supporting weight-related concerns: Dietitians integrated within dental teams sensitively identify, discuss and respond to weight-related concerns with families. Where appropriate, dietitians undertake anthropometric assessment (height and weight measurements and BMI calculated) and initiate referral for further dietetic support. | Training and skill development for integrated oral-systemic health care: Dietitians and dental professionals participate in joint training and skill-development activities that support collaborative oral-systemic dietary care within the clinic. |
| Supporting families to implement oral-health-related dietary advice: Dietitians provide tailored dietary advice to families, supporting them to translate oral-health dietary advice into feasible everyday practices. | Supportive structures for interdisciplinary collaboration: Dietitians and dental professionals establish and participate in structured collaborative activities to support integration and coordinate patient care. |
|  | Communication pathways for collaborative care: Dietitians and dental professionals exchange patient care information using agreed communication systems to facilitate consistent and effective patient care. |
|  | Role clarity and responsibilities within the care pathway: Dietitians and dental professionals define and enact clear professional roles and responsibilities to support coordinated care delivery. |

COM-B, Capability, Opportunity, Motivation- Behaviour; BCW, Behaviour Change Wheel; BMI, body mass index

**Supplementary Table 3. Provisional specification of target behaviours and enabling conditions (pre-Delphi)**

| Target behaviour summary | Who needs to perform the behaviours? | What do they need to do differently? | When and where should the behaviour occur? | How often should the behaviour occur? | With whom should the behaviour occur? |
| --- | --- | --- | --- | --- | --- |
| Sensitive Approaches to Support Weight-Related Concerns  *(Clinical behaviour)* | Dietitians integrated within dental teams. | Identify, discuss and support paediatric weight-related sensitively and initiate dietetic referral within the dental clinic if concerns arise. | Within the dental clinic. | To be determined via Delphi consensus. | With families including paediatric patients and caregivers. |
| Supporting Families to Implement Oral-Health-Related Dietary Advice  *(Clinical behaviour)* | Dietitians (via referral from dental professionals). | Deliver tailored dietary counselling addressing practical, cultural socioeconomic barriers, and link oral and systemic health advice. (Regardless of weight). | In the dental clinic.  To be determined via Delphi consensus. | Referral criteria to be determined via Delphi consensus. | With families including paediatric patients and caregivers.  Interprofessional communication between dental and dietetic teams. |
| Training and Skill Development  *(Enabling condition*) | Dietitians and dental professionals. | Participate in training to improve knowledge for interprofessional working. | To be determined via Delphi consensus. | To be determined via Delphi consensus. | Dietitians and dental professionals. |
| Supportive Structures *(Enabling condition*) | Service leads, local teams, dietitians and dental professionals. | Establish systems (e.g., meetings, referral templates, MDT meetings) to enable coordination. | Embedded within ongoing dental clinic practice. | To be determined via Delphi consensus. | Service leads, local teams, admin staff, dietitians and dental professionals. |
| Communication Pathways  *(Enabling condition*) | Dietitians and dental professionals. | Maintain structured communication and referral feedback processes (e.g. shared electronic records, agreed reporting formats). | To be determined via Delphi consensus. | To be determined via Delphi consensus. | Dietitians, dental professionals, admin teams and families. |
| Role Clarity and Responsibilities  *(Enabling condition*) | Dietitians, dental professionals and service managers. | Define, document and enact clear professional boundaries and responsibilities. | During service planning and ongoing professional development. | Periodically as roles evolve and to be decided in the Delphi. | All members of the multidisciplinary team. |

**Supplementary Table 4. Provisional COM-B mapping (identifying what needs to change) (pre-Delphi)**

|  | *Capability (Psychological)* | *Capability (Physical)* | *Opportunity (social)* | *Opportunity (Physical)* | *Motivation (Reflective)* | *Motivation (Automatic)* |
| --- | --- | --- | --- | --- | --- | --- |
| Sensitive approaches to support weight-related concerns  (*Clinical behaviour)* | Dietitian knowledge of growth monitoring, BMI interpretation, sensitive discussions already established. | Dietitian competent in paediatric anthropometry.  May require weight equipment in the dental clinic. | Supportive dental-dietetic team culture recognising dietitian’s role in weight-related care. | Private space and sufficient time for sensitive conversations. | Recognition of weight-related care which can form part of wider preventive oral care. | Confidence in new integrated role. |
| Supporting families to implement oral-health-related dietary advice  (*Clinical behaviour)* | Dietitian skills in motivational interviewing and behavioural dietary counselling with the ability to related to oral health. | Dietitian ability to tailor dietary advice that considers social determinants. | Collaborative relationship with the dental team to ensure consistent messaging. | Time and systems to allow extended or follow-up consultations. Support from local dietetic teams. | Belief that joint working improves outcomes; professional ownership. | Reinforcement from caregiver success and appreciation. |
| Training and skill development  *(Enabling condition*) | Understanding of oral-systemic health links. | Competence to apply training in real world scenarios. | Culture of shared learning and mutual respect. | Access to joint training and resources. | Commitment to interprofessional learning and self-efficacy. | Professional satisfaction from collaborative efforts over time. |
| Supportive structures  *(Enabling condition)* | Not primary determinant. | Ability to access resources when required. | Trusting relationships within the team. | Systems supporting MDT meetings, shared records, referral guides. | Value placed on integrated care and collective responsibility. | Habitual collaboration reinforced by improved efficiency and outcomes. |
| Communication pathways  *(Enabling condition)* | Knowledge of communication protocols and data sharing standards. | Skill in using shared referral and communication systems. | To be determined via Delphi consensus. | Availability of interoperable systems and administrative support. | Not primary determinant. | Routine communication behaviour reinforced through workflow repetition. |
| Role clarity and responsibility  *(Enabling condition)* | Clear understanding of own and others professional scope. | Ability to apply role boundaries confidently. | Shared understanding of interprofessional accountability. | Structured governance processes and documentation. | Professional identity aligned with care pathway. | Sense of ownership and security as roles stabilises over time. |

**Supplementary Table 5. Round 1 Questions**

| Target behaviour/ enabling condition | Question |
| --- | --- |
| Sensitive approaches to supporting weight-related concerns | Under what circumstances, if any, do you think the dietitian should conduct height and weight measurements to calculate Body Mass Index* ~BMI~ (within the dental clinic)? |
| Sensitive approaches to supporting weight-related concerns | If you do not think height and weight measurements are appropriate in dental clinics, how else, if at all, could dietitians identify and support children with growth and weight concerns in this setting? |
| Sensitive approaches to supporting weight-related concerns | When, if at all, do you think height and weight measurements would be most appropriate, for the dietitian to conduct during the dental visit? |
| Sensitive approaches to supporting weight-related concerns | How frequently, if at all, should the dietitian conduct height and weight measurements (within the dental clinic) to ensure they are effective and appropriate? |
| Sensitive approaches to supporting weight-related concerns | What specific strategies or tools could help ensure height and weight assessments (if at all) are conducted in a sensitive, non-stigmatising way? |
| Supporting families to implement oral-health-related dietary advice | What information should be included in a referral from a dental professional to a dietitian to support effective implementation of dietary support? |
| Supporting families to implement oral-health-related dietary advice | At what point, if any, should a child be referred to a dietitian for oral-health-related dietary support within the integrated care pathway? |
| Role clarity and responsibility | In your opinion, how should dietitians working in paediatric dental clinics manage complex cases where children are already under specialist care or have medical conditions requiring advanced dietary support? |
| Training and skill development | What key topics should be included in training for dental professionals and dietitians to effectively deliver an integrated care pathway for supporting paediatric oral health, growth, and dietary needs?  What training formats do you think would be most effective and realistic for busy healthcare professionals working in dental or dietetic roles? |
| Training and skill development | How do you think training programmes should balance individual learning (e.g., dietitian-specific or dental-specific skills) with joint interprofessional activities? |
| Supportive structures | What kinds of support structures would best enable effective collaboration between dietitians and dental professionals within an integrated care model? |
| Supportive structures | What additional support would dietitians need to succeed in a paediatric dental clinic, especially when working across oral and systemic health concerns? |
| Communication pathways | How frequently should collaborative team meetings or support reviews take place between dental professionals and dietitians to ensure effective care coordination? |
| Communication pathways | What should be the primary method of communication between dietitians and dental professionals when working together in a shared care model? |
| Communication pathways | How often do you think dietitians and dental professionals should communicate about shared patients to maintain continuity of care? |
| Communication pathways | What specific types of information should be shared between dietitians and dental professionals to support joined-up, consistent patient care? |
| Role clarity and responsibility | Could there be any overlap between the roles of dietitians and dental professionals working within the same setting? If so, where might that happen and how could it be avoided? |

*Supplementary Results*

**Supplementary Figure 2. Additional demographic information**

Supplementary Figure 2.1. Years of professional experience among experts completing Delphi Round 3

Supplementary Figure 2.2. Age distribution among experts completing Delphi Round 3

Supplementary Figure 2.3. Highest level of education among experts completing Delphi Round 3

| **Supplementary Table 6. Full consensus statements**  Statements shown in black reached full consensus in Round 2, while statements shown in blue reached full consensus in Round 3.  Question | Consensus reached | Overall consensus-  Full-panel | Direction | Dietitian Consensus | Dental Professional  Consensus |
| --- | --- | --- | --- | --- | --- |
| What specific strategies or tools could help ensure height and weight assessments are conducted in a sensitive, non-stigmatising way in the dental clinic? | | | | | |
| 7.1 Clearly explain the purpose of measurement to families | ✓ | 89% | Agreement | 92% | 87% |
| 7.2 Frame BMI as part of holistic care, not weight-focused messaging | ✓ | 93% | Agreement | 100% | 87% |
| 7.3 Emphasising the link between oral health and growth can help families understand the relevance of BMI | ✓ | 88% | Agreement | 100% | 77% |
| 7.4 Conversations about weight and growth should use neutral, supportive, non-judgemental language | ✓ | 100% | Agreement | 100% | 100% |
| 7.5 The language used should be tailored to the child’s age, developmental stage, and emotional needs | ✓ | 100% | Agreement | 100% | 100% |
| 7.7 Visual aids (e.g., growth charts, BMI percentiles) should be used to explain results to families | ✓ | 86% | Agreement | 92% | 80% |
| 7.8 Short, age-appropriate videos or animations can help children understand the importance of healthy growth | ✓ | 76% | Agreement | 75% | 77% |
| 7.9 Families should be informed in advance that height and weight measurement may occur, with an option to decline | ✓ | 93% | Agreement | 85% | 100% |
| 7.10 Written materials (e.g., appointment letters or pre-visit leaflets) should explain what to expect and why it is being done | ✓ | 93% | Agreement | 92% | 93% |
| 7.11 The referral criteria and process should be clearly defined (e.g., routine measurements, concerns from dental professionals or caregivers) to prevent families from feeling ‘singled out’ | ✓ | 89% | Agreement | 77% | 100% |
| 7.12 Height and weight measurements should be conducted in a private room | ✓ | 93% | Agreement | 100% | 87% |
| 7.13 The environment should be child-friendly and welcoming (e.g., posters, bright colours, reward stickers) | ✓ | 96% | Agreement | 92% | 100% |
| 7.14 Dietitians, not dental staff, should lead any conversations about growth, weight, or BMI results | ✓ | 80% | Agreement | 75% | 85% |
| 7.15 Dental staff should receive training on how to respond to caregivers if they ask about weight measurements | ✓ | 96% | Agreement | 100% | 93% |
| 7.16 Framing weight measurements as an opportunity for a growth and health assessment with a positive narrative | ✓ | 86% | Agreement | 100% | 73% |
| At what point should a child be referred to a dietitian for dietary support based on oral health-related concerns as part of the integrated care pathway? | | | | | |
| 9.4 Referral should occur if oral symptoms affect eating | ✓ | 82% | Agreement | 92% | 73% |
| 9.5 Referral should happen after repeated dental dietary advice fails or is not implemented | ✓ | 84% | Agreement | 75% | 92% |
| 9.7 A child should be referred if the family specifically requests support, regardless of disease severity | ✓ | 75% | Agreement | 77% | 73% |
| 9.8 Referral should occur when families express confusion about or difficulty implementing dental dietary advice | ✓ | 82% | Agreement | 85% | 80% |
| What information should be included in a referral from a dental professional to a dietitian to support effective implementation of dietary support? (Beyond basic patient details like name, birth date, etc.) | | | | | |
| 10.2 Growth concerns (e.g., faltering growth, overweight) should be shared to guide both dietary and oral health support | ✓ | 75% | Agreement | 77% | 73% |
| 10.3 Relevant medical history (including medications) | ✓ | 96% | Agreement | 92% | 100% |
| 10.4 Oral health findings (e.g., caries, pain, erosion, ability to eat secondary to oral pain) and any suspected dietary causes, including caries risk assessment | ✓ | 93% | Agreement | 100% | 87% |
| 10.5 Information on oral health progression (e.g., stable, improving, worsening) | ✓ | 100% | Agreement | 100% | 100% |
| 10.6 Information on prior dietary advice and engagement with advice | ✓ | 96% | Agreement | 100% | 93% |
| 10.7 Dental appointment attendance | ✓ | 79% | Agreement | 85% | 73% |
| 10.8 Caregivers motivation, concerns, and readiness to change | ✓ | 86% | Agreement | 92% | 80% |
| 10.11 Family or social context (e.g., food insecurity, cooking facilities) impacting dietary behaviours | ✓ | 76% | Agreement | 75% | 77% |
| 10.12 Additional needs (e.g., autism spectrum disorder, sensory issues, neurodiversity) | ✓ | 96% | Agreement | 100% | 93% |
| 10.13 Any safeguarding concerns flagged through appropriate channels | ✓ | 96% | Agreement | 100% | 93% |
| In your opinion, how should dietitians working in paediatric dental clinics manage complex cases where children are already under specialist care or have medical conditions requiring advanced dietary support? | | | | | |
| 11.1 When a child is already under a specialist dietitian, the dietitian in the dental clinic should not be involved in providing any nutritional care | ✓ | 75% | Neutral | 77% | 73% |
| 11.2 When a child is already under specialist care, the dietitian in the dental clinic should liaise with the existing care team before deciding whether to stay involved | ✓ | 89% | Agreement | 92% | 87% |
| 11.3 The dietitian in the dental clinic may remain involved after liaising with the existing team if their input is complementary | ✓ | 82% | Agreement | 85% | 80% |
| 11.4 Dietitians in dental clinics should act as bridge between primary care, community and hospital teams, providing light-touch support between specialist reviews | ✓ | 79% | Agreement | 77% | 80% |
| 11.5 The dietitian in the dental clinic should provide first-line advice, not work outside their scope of practice and should refer on to specialist services as soon as cases appear more complex | ✓ | 86% | Agreement | 77% | 93% |
| 11.8 For children with medical needs affecting diet (e.g., autism spectrum disorder, diabetes, etc.) the dietitian in the dental clinic should support oral health focused advice within their scope | ✓ | 89% | Agreement | 85% | 93% |
| How do you think training programmes should balance individual learning (e.g., dietitian-specific or dental-specific skills) with joint interprofessional activities? | | | | | |
| 13.3 Training should be separate for both professions the whole time | ✓ | 84% | Disagreement | 92% | 77% |
| What key topics should be included in training for dental professionals and dietitians to effectively deliver an integrated care pathway for supporting paediatric oral health, growth, and dietary needs? | | | | | |
| 14.1 Diet-oral health links | ✓ | 96% | Agreement | 100% | 93% |
| 14.2 Nutrient content of food and drink and how these affect oral health | ✓ | 96% | Agreement | 100% | 93% |
| 14.3 Cariogenic mechanisms (e.g., oral microbiome, frequency of sugar intake, saliva function) | ✓ | 93% | Agreement | 100% | 87% |
| 14.4 The impact of medical conditions on oral health and diet (e.g., reflux, diabetes, food allergies) | ✓ | 96% | Agreement | 100% | 93% |
| 14.5 Understanding how to realistically and practically balance oral health advice and nutritional needs, especially in children with restricted diets or oral nutritional supplement (ONS) use | ✓ | 100% | Agreement | 100% | 100% |
| 14.6 Oral health risks in medically vulnerable children (e.g., infection risk in immunocompromised children) | ✓ | 96% | Agreement | 92% | 100% |
| 14.7 Behaviour change strategies to help families implement advice effectively | ✓ | 96% | Agreement | 100% | 93% |
| 14.8 Sensitive communication about weight, growth, and dietary habits, including stigma-free language | ✓ | 96% | Agreement | 100% | 93% |
| 14.9 Ethical considerations in shared care, including consent, confidentiality, and professional boundaries | ✓ | 96% | Agreement | 100% | 93% |
| 14.10 Practical strategies for educating families, including visual aids and developmentally appropriate messaging | ✓ | 96% | Agreement | 100% | 93% |
| 14.11 Understanding the role of mental health services and how to collaborate for children under child and adolescent mental health services or eating disorder teams | ✓ | 86% | Agreement | 92% | 80% |
| 14.12 Tailoring advice for families from different cultural and socioeconomic backgrounds | ✓ | 93% | Agreement | 100% | 87% |
| 14.13 Working with children with learning disabilities, autism, Avoidant/restrictive food intake disorder, or sensory-based eating needs | ✓ | 93% | Agreement | 92% | 93% |
| 14.14 Awareness of school meals, social food norms, and their impact on diet in different age groups | ✓ | 80% | Agreement | 83% | 77% |
| 14.15 Principles of interprofessional teamwork and communication, including shared records and feedback loops | ✓ | 93% | Agreement | 100% | 87% |
| 14.16 Pathways for referral to specialist services, including local dietitians, mental health teams, and safeguarding leads | ✓ | 100% | Agreement | 100% | 100% |
| What training formats do you think would be the most effective and practical for busy healthcare professionals in dental or dietetic roles? | | | | | |
| 15.2 Live virtual workshops to offer opportunities for case discussion without requiring travel | ✓ | 84% | Agreement | 83% | 85% |
| 15.3 At least one in-person workshop should be included to facilitate networking and deeper connections. | ✓ | 96% | Agreement | 100% | 93% |
| 15.5 Multidisciplinary Team clinical supervision sessions to explore integrated case studies and shared learning | ✓ | 88% | Agreement | 100% | 77% |
| 15.7 Integrating case discussions into team meetings or clinical huddles | ✓ | 86% | Agreement | 92% | 80% |
| 15.8 A hybrid approach, combining self-paced online modules with in-person workshops and/or clinical supervision sessions | ✓ | 82% | Agreement | 85% | 80% |
| What support structures are most important to enable collaboration between dietitians and dental professionals in an integrated care model? | | | | | |
| 16.4 Annual review meetings to evaluate service impact | ✓ | 80% | Agreement | 75% | 85% |
| 16.6 Joint CPD workshops | ✓ | 75% | Agreement | 77% | 73% |
| 16.10 Structured feedback systems for outcomes and referrals | ✓ | 75% | Agreement | 77% | 73% |
| 16.11 Use of shared care plans or communication templates | ✓ | 79% | Agreement | 77% | 80% |
| 16.12 Clear, localised referral criteria and pathways | ✓ | 96% | Agreement | 92% | 100% |
| 16.13 Shared digital notes systems accessible to both professions | ✓ | 89% | Agreement | 92% | 87% |
| 16.14 Integration of communication into existing clinical systems | ✓ | 89% | Agreement | 85% | 93% |
| 16.15 Interdisciplinary training which is CPD accredited for both professions | ✓ | 93% | Agreement | 85% | 100% |
| What additional support would dietitians need to succeed in a paediatric dental clinic, especially when working across oral and systemic health concerns? | | | | | |
| 17.1 Access to community and hospital medical records and growth charts | ✓ | 84% | Agreement | 92% | 77% |
| 17.3 Connection to specialist dietetic teams (e.g., paediatric, eating disorders) | ✓ | 89% | Agreement | 92% | 87% |
| 17.4 The ability to attend local dietetic department meetings | ✓ | 80% | Agreement | 75% | 85% |
| 17.5 Dietitians should be linked to dental networks and communication forums for regular interprofessional updates | ✓ | 88% | Agreement | 83% | 92% |
| 17.6 Shadowing dental professionals to understand clinical context | ✓ | 79% | Agreement | 85% | 73% |
| 17.7 Training in dental terminology and treatment pathways | ✓ | 82% | Agreement | 77% | 87% |
| 17.8 Structured induction to the dental clinic | ✓ | 79% | Agreement | 85% | 73% |
| 17.9 Mentorship from senior dietitians | ✓ | 79% | Agreement | 85% | 73% |
| 17.10 Point of contact in the dental team for clinical queries (a dental supervisor) | ✓ | 86% | Agreement | 85% | 87% |
| 17.14 Allow time to build rapport, interprofessional relationships and respect between professionals | ✓ | 89% | Agreement | 92% | 87% |
| How frequently should collaborative team meetings or support reviews take place between dental professionals and dietitians to ensure effective care coordination? | | | | | |
| 18.10 Annual review meetings for evaluation | ✓ | 75% | Agreement | 77% | 73% |
| 18.13 Communication between meetings - twice weekly updates during periods of active involvement from the dietitian in the dental clinic | ✓ | 84% | Neutral | 83% | 85% |
| 18.14 Communication between meetings - When significant changes occur in care | ✓ | 82% | Agreement | 77% | 87% |
| 18.15 Communication between meetings - ongoing documentation in shared notes | ✓ | 75% | Agreement | 77% | 73% |
| 18.16 Communication between meetings - use of flagging systems to alert to dietary issues | ✓ | 75% | Agreement | 77% | 73% |
| What should be the primary method of communication between dietitians and dental professionals when working together in a shared care model? | | | | | |
| 19.1 Shared digital patient records | ✓ | 93% | Agreement | 92% | 93% |
| What strategies are most important to reduce role overlap and confusion between dietitians and dental professionals? | | | | | |
| 20.1 Clear role boundaries | ✓ | 86% | Agreement | 85% | 87% |
| 20.2 Dental professionals should focus on providing brief oral health–focused dietary advice (e.g., sugar frequency, bottle use), while dietitians deliver more in-depth | ✓ | 79% | Agreement | 85% | 73% |
| 20.3 There should be a shared understanding across the team about when to escalate from basic dietary advice to a specialist dietetic referral | ✓ | 93% | Agreement | 92% | 93% |
| 20.4 Referral criteria or flowcharts to guide escalation | ✓ | 96% | Agreement | 100% | 93% |
| 20.5 Shared understanding of when to refer to dietetics | ✓ | 96% | Agreement | 100% | 93% |
| 20.6 Documenting dietary advice in shared records | ✓ | 93% | Agreement | 92% | 93% |
| 20.7 Consistent and non-conflicting dietary messages between teams | ✓ | 93% | Agreement | 100% | 87% |
| 20.8 All dietary advice provided by the dental team should align with the broader care plan agreed with the dietitian. | ✓ | 86% | Agreement | 100% | 73% |
| 20.9 Dental professionals should not conduct full dietary assessments or provide nutritional counselling unless trained to do so | ✓ | 82% | Agreement | 92% | 73% |
| 20.11 Clear communication with families about roles | ✓ | 93% | Agreement | 92% | 93% |
| 20.12 Co-created patient materials explaining professional responsibilities | ✓ | 79% | Agreement | 77% | 80% |
| 20.13 Joint training to align approaches | ✓ | 96% | Agreement | 100% | 93% |

**Supplementary Table 7. Subgroup-level exploratory consensus statements informing weight-related care**

This supplementary file presents follow-up statements that reached sub-group-level exploratory consensus (≥70%) within model-specific subgroups for the weight-related model-choice item.

These statements were derived from analyses restricted to participants who selected the most frequently endorsed model in Round 3 (combined anthropometric and non-anthropometric approach; n=10). Statements are presented to support transparency and to distinguish exploratory preferences from full panel consensus

Supplementary Table 7.1: Exploratory consensus statements related to anthropometric measurement

| Statement | % Agreement (subgroup) | Notes |
| --- | --- | --- |
| BMI should be measured at new patient registration | 70% | Exploratory |
| BMI should not be repeated if recent measurements are available (e.g., National Childhood Measurement Programme, GP) | 70% | Exploratory |
| Children with medical conditions affecting growth should be monitored more frequently | 100% | Exploratory |

Supplementary Table 7.2: Exploratory consensus statements related to non-anthropometric referral criteria

| Statement | % Agreement (subgroup) | Notes |
| --- | --- | --- |
| Caregiver expresses concern about growth, weight, or eating | 90% | Exploratory |
| Dental professional observes visible signs of underweight, overweight or obesity | 100% | Exploratory |
| Dental professional identifies weight concern risk factors through dietary assessment | 80% | Exploratory |
| Results from a nutrition screening questionnaire indicate risk of unhealthy body weight | 80% | Exploratory |
| The child expresses concern about their body or weight | 80% | Exploratory |
| The child has a medical condition affecting growth or nutrition | 90% | Exploratory |
| A recent measurement from GP or National Childhood Measurement Programme (NCMP) shows rapid change | 100% | Exploratory |

**Supplementary Table 8. Refined COM-B mapping (identifying what needs to change) following Delphi consensus**

This table presents a refined COM-B mapping based on Delphi consensus findings. Determinants reflect agreed components of behaviours/enabling conditions, while elements not directly addressed in the Delphi are not discussed

|  | *Capability (Psychological)* | *Capability (Physical)* | *Opportunity (social)* | *Opportunity (Physical)* | *Motivation (Reflective)* | *Motivation (Automatic)* |
| --- | --- | --- | --- | --- | --- | --- |
| Sensitive approaches to support weight-related concerns  (*Clinical behaviour)* | Dietitians require capability to interpret anthropometric and non-anthropometric indicators and to conduct sensitive, stigma-aware discussions. However, the specific approach to identifying concerns remains uncertain and may involve multiple strategies. | Dietitian competent in paediatric anthropometry and dietary assessment, with flexibility in how these are applied. | Recognition and acceptance of the dietitian’s role in addressing weight-related concerns within the dental team. | Access to appropriate space, time and clinical information; opportunity to use different identification of concerns approach. | Not explicitly identified in Delphi findings. | Not explicitly identified in Delphi findings. |
| Supporting families to implement oral-health-related dietary advice  (*Clinical behaviour)* | Dietitian skills in motivational interviewing and behavioural dietary counselling with the ability to related to oral health. | Dietitian skills in delivering personalised dietary counselling and applying behaviour change strategies (in relation to oral health). | Collaborative relationships enabling consistent messaging between professionals. | Access to referral pathways, and availability of dietetic services. | Not explicitly identified in Delphi findings. | Not explicitly identified in Delphi findings. |
| Training and skill development  *(Enabling condition*) | Understanding of oral-systemic health links. | Ability to apply training in practice. | Culture of shared learning and mutual respect. | Access to joint training, supervision and learning resources. | Not explicitly identified in Delphi findings | Not explicitly identified in Delphi findings |
| Supportive structures  *(Enabling condition)* | Not primary determinant | Ability to use systems supporting collaboration (e.g. referral pathways, communication systems) | Trusting relationships and time to develop such. | Systems enabling collaboration (shared records, access to medical records, mentorship structures). | Not explicitly identified in Delphi findings | Not explicitly identified in Delphi findings |
| Communication pathways  *(Enabling condition)* | Knowledge of communication protocols and data sharing standards. | Skill in using shared referral and communication systems. | Agreed communication norms | Availability of shared digital systems. | Not explicitly identified in Delphi findings. | Not explicitly identified in Delphi findings |
| Role clarity and responsibility  *(Enabling condition)* | Clear understanding of own and others professional scope. | Ability to apply role boundaries in practice. | Shared understanding of responsibilities and accountability. | Structured governance processes and documentation. | Not explicitly identified in Delphi findings | Not explicitly identified in Delphi findings |

**References**

1. Michie S, Atkins L, West R. *The Behaviour Change Wheel: A Guide to Designing Interventions*. Silverback Publishing; 2014. www.behaviourchangewheel.com

2. Authors removed for anonymisation. Title removed for anonymisation.

3. O’Brien BC, Harris IB, Beckman TJ, Reed DA, Cook DA. Standards for reporting qualitative research: a synthesis of recommendations. *Acad Med*. 2014;89(9):1245-1251. doi:10.1097/ACM.0000000000000388

4. Franki J, Hayes MJ, Taylor JA. The provision of dietary advice by dental practitioners: a review of the literature. *Community Dent Health*. 2014;31(1):9-14.

5. Hayes MJ, Franki J, Taylor JA. The Frequency of Dietary Advice Provision in a Dental Hygiene Clinic: A Retrospective Cross-Sectional Study. *J Dent Hyg*. 2016;90(1):12-17.

6. Large JF, Madigan C, Graham H, Biddle GJH, Sanders J, Daley AJ. Public and dental teams’ views about weight management interventions in dental health settings: Systematic review and meta-analysis. *Obesity Reviews*. 2024;25(6):e13726. doi:10.1111/obr.13726

7. More FG, Sasson LM, Godfrey EM, Sehl RB. Collaboration Between Dietetics and Dentistry: Dietetic Internship in Pediatric Dentistry. *Top Clin Nutr*. 2005;20(3):259-268.

8. Fernandez JB, Ahearn K, Atar M, et al. Interprofessional Educational Experience Among Dietitians After a Pediatric Dentistry Clinical Rotation. *Topics in Clinical Nutrition*. 2017;32(3):193.

9. Stager SC, Levine AM. The need for nutritionists: A survey of dental practitioners. *Journal of the American Dietetic Association*. 1990;90(1):100-102. doi:10.1016/S0002-8223(21)01473-5

10. Dietitians Australia. *Joint Position Statement on Interdisciplinary Collaboration between Accredited Practising Dietitians, Nutrition and Oral Health Professionals for Oral Health and Nutrition*. 2021. https://www.dhsv.org.au/__data/assets/pdf_file/0011/155378/FINAL-VERSION-JPS-OH_Nutrition-Collab_20062022.pdf

11. Ong HH, Wan CCJWJ, Gao X. Interprofessional Collaboration in Addressing Diet as a Common Risk Factor: A Qualitative Study. *Journal of Research in Interprofessional Practice and Education*. 2015;5(2). doi:10.22230/jripe.2015v5n2a215

12. Michie S, van Stralen MM, West R. The behaviour change wheel: A new method for characterising and designing behaviour change interventions. *Implementation Science*. 2011;6(1):42. doi:10.1186/1748-5908-6-42

13. NHS. *Integrated Care Systems: Design Framework*. 2021. Accessed October 31, 2025. https://www.england.nhs.uk/wp-content/uploads/2021/06/B0642-ics-design-framework-june-2021.pdf

14. Lieffers JRL, Vanzan AGT, Rover de Mello J, Cammer A. Nutrition Care Practices of Dietitians and Oral Health Professionals for Oral Health Conditions: A Scoping Review. *Nutrients*. 2021;13(10):3588. doi:10.3390/nu13103588

15. *What Is a Dietitian?* 2020. Accessed March 12, 2025. https://www.youtube.com/watch?v=4RmLhe72Htc

16. Valentijn PP, Schepman SM, Opheij W, Bruijnzeels MA. Understanding integrated care: a comprehensive conceptual framework based on the integrative functions of primary care. *Int J Integr Care*. 2013;13:e010. doi:10.5334/ijic.886
